# FPR2: A Novel Promising Target for the Treatment of Influenza

**DOI:** 10.3389/fmicb.2017.01719

**Published:** 2017-09-05

**Authors:** Marie-Christine Alessi, Nicolas Cenac, Mustapha Si-Tahar, Béatrice Riteau

**Affiliations:** ^1^Aix Marseille Univ, INSERM, INRA, NORT, UMR 1260/1062 Marseille, France; ^2^IRSD, INSERM, INRA, INP-ENVT, Université de Toulouse 3 Toulouse, France; ^3^INSERM, Université de Tours, Centre d'Étude des Pathologies Respiratoires, UMR 1100 Tours, France

**Keywords:** FPR2, formyl peptide receptor, influenza, human, inflammation mediators, antiviral agents

## Abstract

The Formyl-peptide receptor-2 (FPR2) is a seven transmembrane G protein-coupled receptor, which plays an important role in sensing of bacteria and modulation of immune responses. FPR2 is also used by viruses for their own profit. Annexin A1, one of the multiple ligands of FPR2, is incorporated in the budding virus membrane of influenza A viruses (IAV). Thereby, once IAV infect a host cell, FPR2 is activated. FPR2-signaling leads to an increase in viral replication, a dysregulation of the host immune response and a severe disease. Conversely, experiments using FPR2 antagonists in a preclinical model of IAV infections in mice showed that blocking FPR2 protects animals from lethal infections. Thus, FPR2 represents a very attractive host target against influenza. In this review we will give an overview on the pathogenesis of influenza with a focus on the role of FPR2 and we will discuss the advantages of using FPR2 antagonists to treat the flu.

## Introduction

Influenza virus infection is one of the most important infectious diseases affecting the respiratory tract (Palese and Shaw, 2007). Influenza outbreaks are usually associated with mild symptoms, but can also result in millions of cases of severe illness leading to pneumonia, especially among the elderly and young children. Globally, influenza still accounts for 250,000–500,000 deaths every winter (Palese and Shaw, 2007). The etiological agents of the disease are the negative sense and single-stranded RNA influenza viruses, which belong to the family of enveloped viruses. They are classified into four types (A, B, C, and D), of which influenza A viruses (IAV) are the most devastating (Kuiken et al., 2012; Ferguson et al., 2016). IAV are also divided into subtypes according to their hemagglutinin (HA) and neuraminidase (NA) surface proteins. Eighteen different HA and eleven different NA proteins have been described. Many combinations of HA and NA exist but only some virus strains (i.e., H1N1 and H3N2) circulate among humans. Other subtypes can also infect humans but these viruses are hardly, if not at all transmissible from human to human. For example, H7N9 and H5N1 viruses can be transmitted from the avian reservoir directly to humans but do not spread between humans (Imai et al., 2013; Richard et al., 2013).

Upon influenza virus infection, the host immune response is activated in order to limit viral replication and to eliminate infected cells. The innate response is the first line of defense initiated by the recognition of pathogen-associated molecular patterns (PAMP), which in majority are nucleic acids that are not typically present in the host cells (Yoo et al., 2013). Recognition of PAMP occurs through activation of pattern-recognition receptors (PRRs), which include nucleotide-binding oligomerization domain (NOD)-like receptors, Toll-like receptors and retinoic acid-inducible gene-I (RIG)-like helicases. The main specific sensors activated by influenza viruses are TLR3/7/8, NLRP3 and RIG-I. TLR3/7/8 and RIG-1 trigger NFκB and interferon regulated factor 3 (IRF3) transcription factors allowing the synthesis and production of pro-inflammatory cytokines and interferon. NLRP3 inflammasome allows the maturation and release of IL-1β and IL-18 (Berri et al., 2014a). While interferons are the major cytokines that inhibit viral replication, influenza viruses evolved sophisticated strategies to reduce their release (Garcia-Sastre, 2011). The secretion of pro-inflammatory cytokines and chemokines attracts and activates innate immune cells such as natural killer cells, neutrophils and macrophages, that can also afford an effective protection by eliminating influenza virus-infected cells (Kuiken et al., 2012). However, during severe influenza, the virus is inefficiently eliminated, inflammation is excessive and altogether, this results in lung insult and deterioration of the clinical outcome of the infected patients (Peiris et al., 2010; Kuiken et al., 2012). Thus, the main factors contributing to a severe disease are the high capacity of influenza virus to replicate and a dysregulated harmful innate immune response.

## Current available treatments against influenza

To date, two classes of anti-influenza drugs are available: the inhibitors of neuraminidase (zanamivir and oseltamivir) and those of M2 (amantadine and rimantadine) (Ison, 2011). Regarding the viral M2 protein, it is a proton selective channel, necessary for viral replication. M2 forms tetramers and is expressed at the plasma membrane of infected cells. After entry into the cell, the virion is located in endosomes, where the low pH activates the M2 channel permitting proton flux and acidification of the interior of the virion. This acidification dissociates the viral RNA from its bound matrix proteins and is thus an important step for the release of the virus genome from the endosome to the cytoplasm (Helenius, 1992). In addition, M2 has a role in virus assembly, budding and morphogenesis (Rossman et al., 2010a,b). Amantadine and rimantadine are FDA (Food and Drug Administration)-approved drugs binding to M2 and blocking its function. Unfortunately, influenza B viruses are naturally insensitive to M2 ion channel blockers since BM2, the ion channel protein of these viruses has almost no sequence homology with the M2 of IAV. Regarding IAV, they have acquired subtype wide resistance mutations over time (Pontoriero et al., 2008). Thus, these drugs do not work against influenza B and most types of IAV. To date, FDA does not recommend their use and these antiviral molecules are not commercialized anymore in western countries. Nowadays, the market for influenza treatments is thus dominated by the NA inhibitors.

Regarding the viral NA, it plays a critical role for virus transmission from cell to cell. The first step of the influenza virus replication cycle is the binding of the virion to the host cell. This occurs through the interaction of the viral HA protein with sialic acids on the membrane of the host cell. The NA is a glycoside hydrolase enzyme that removes influenza virus receptor-binding sites and enables the newly synthesized virions to detach from the infected cells at the end of the viral life cycle. It also prevents virus self-aggregation. Thus, virions grown in the presence of NA inhibitors form aggregates near the cell surface, preventing virus spread (Palese and Compans, 1976). The drugs zanamivir (Relenza; Glaxo Smith Kline) and oseltamivir (Tamiflu; Roche) are FDA approved for the treatment and prevention of uncomplicated acute influenza illness. The recommended duration of treatment is 5 days, which allows a reduction of the severity of the symptoms but only if taken early enough after infection. In average, NA inhibitors reduce the duration of symptoms by 1 day if treatment is started within 24–48 h after symptoms begin. Oseltamivir treatment is associated with nausea and vomiting (Treanor et al., 2000) and in some cases, more significant side effects, such as psychiatric events, were described (Jefferson et al., 2014). The viral NA protein has a high mutation rate and influenza viruses also achieve resistance to drugs that target NA, without affecting their virulence (Hay and Hayden, 2013; van der Vries et al., 2013). In majority, mutations that conferred viral resistance were a substitution of a histidine to a tyrosine at residue 274 of the NA.

Altogether, our current available drugs against influenza target viral proteins and have the disadvantage to face virus resistance. Thus, to overcome this resistance challenge (Ison, 2011; van der Vries et al., 2013), active research has been developed to find novel molecules targeting the host instead of the virus to limit the selection pressure on influenza viruses (Ludwig, 2011; Planz, 2013). As an example DAS181, a recombinant sialidase, which prevents IAV binding to the host cells, has been evaluated in phase II clinical trials. Treatment with DAS181 diminished viral loads in infected patients, but no improvements in influenza symptoms were observed (Moss et al., 2012). Thus, although host-directed antivirals are novel promising approaches, immune modulatory compounds that will both prevent viral replication and temper inflammation, such as those targeting immune receptors or signaling pathways (Khoufache et al., 2009; Haasbach et al., 2017), might offer a better perspective regarding the amelioration of the clinical symptoms.

## FPR2: a checkpoint receptor involved in inflammatory processes

Formyl peptide receptors (FPR) are a family of seven transmembrane domains receptors coupled to G protein. While three different FPR were described in humans (FPR1-3), at least eight FPR exist in mice (mFPR), designated FPR1 (FPR1), FPR-rs1 (FPR3 or LXA4 receptor), FPR-rs2 (FPR2), FPR-rs3, FPR-rs4, FPR-rs5, FPR-rs6, and FPR-rs7 (Gao et al., 1998; Wang and Ye, 2002). While no counterparts in human were described for FPR-rs3-7, the mouse ortholog of FPR1 is encoded by mouse FPR1 based on sequence similarities and affinity of fMPL ligand binding (He et al., 2013). Regarding FPR-rs2 (FPR2) and FPR-rs1 (FPR3), they are most likely the orthologs of human FPR2 as those receptors bind LXA4, in contrast to the others (Takano et al., 1997; Vaughn et al., 2002). In addition human and mouse FPR2 show 76% amino acid identity (Courtin et al., 2017).

In humans, FPR2 (or FPRL1/ALX) is expressed by many cells, including epithelial and endothelial cells, fibroblasts, most if not all immune cells as well as neuronal cells. It binds several kinds of ligands (He and Ye, 2017), i.e., it is activated by chemotactic formyl peptides (products of bacteria or derived from the mitochondria), bioactive lipid metabolites of arachidonic acid or docohexanoic acid (lipoxin A4 and resolvin D1, respectively), as well as the cellular Annexin A1 protein or urokinase-type plasminogen activator receptor.

Initially recognized as a pattern recognition receptor, which detects bacterial microorganisms through formylated peptides, FPR2 elicits pro-inflammatory responses. *In vitro*, FPR2 activation promotes inflammatory responses and increases monocytes chemotaxis and neutrophils recruitment (Carp, 1982; De et al., 2000). *In vivo*, mice deficient in FPR2 display increased susceptibility to *Listeria monocytogenes*, increased bacteria load in the liver and reduced neutrophils infiltration (Liu M. et al., 2012). In addition, it was demonstrated that FPR2 activation elicit pro-inflammatory responses upon activation by serum amyloid A (Ye et al., 2009). These reports highlight the role of FPR2 in promoting an inflammatory response. However, a distinct function for FPR2 is also to inhibit and resolve inflammation. During acute inflammation, FPR2 is activated by anti-inflammatory lipid mediators such as lipoxin A4 (LXA_4_), resolvin D1 or the glucocorticoid-modulated protein Annexin A1, allowing a resolution of inflammation and return to homeostasis. The concept that inflammation resolves through active processes is now commonly accepted (Serhan and Savill, 2005), and FPR2 plays a key role in this process (Perretti et al., 2002; Chiang et al., 2006; Perretti and D'Acquisto, 2009). Altogether, FPR2 is thus emerging as a central checkpoint receptor in inflammatory processes, although its pro vs. anti-inflammatory functions are not well understood. Most likely, the versatile function of FPR2 is dependent on the accessible ligands available, in a spatio-temporal manner. While the pro-inflammatory potential of formylated peptides would be elevated early upon injury/infection due to the presence of necrotic cells or bacteria, the generation of anti-inflammatory mediators such as LXA_4_ or resolvin D1 may become more prominent at later stages, during inflammation resolution.

## Activation of FPR2 by IAV and deterioration of the disease

**Figure 1:**
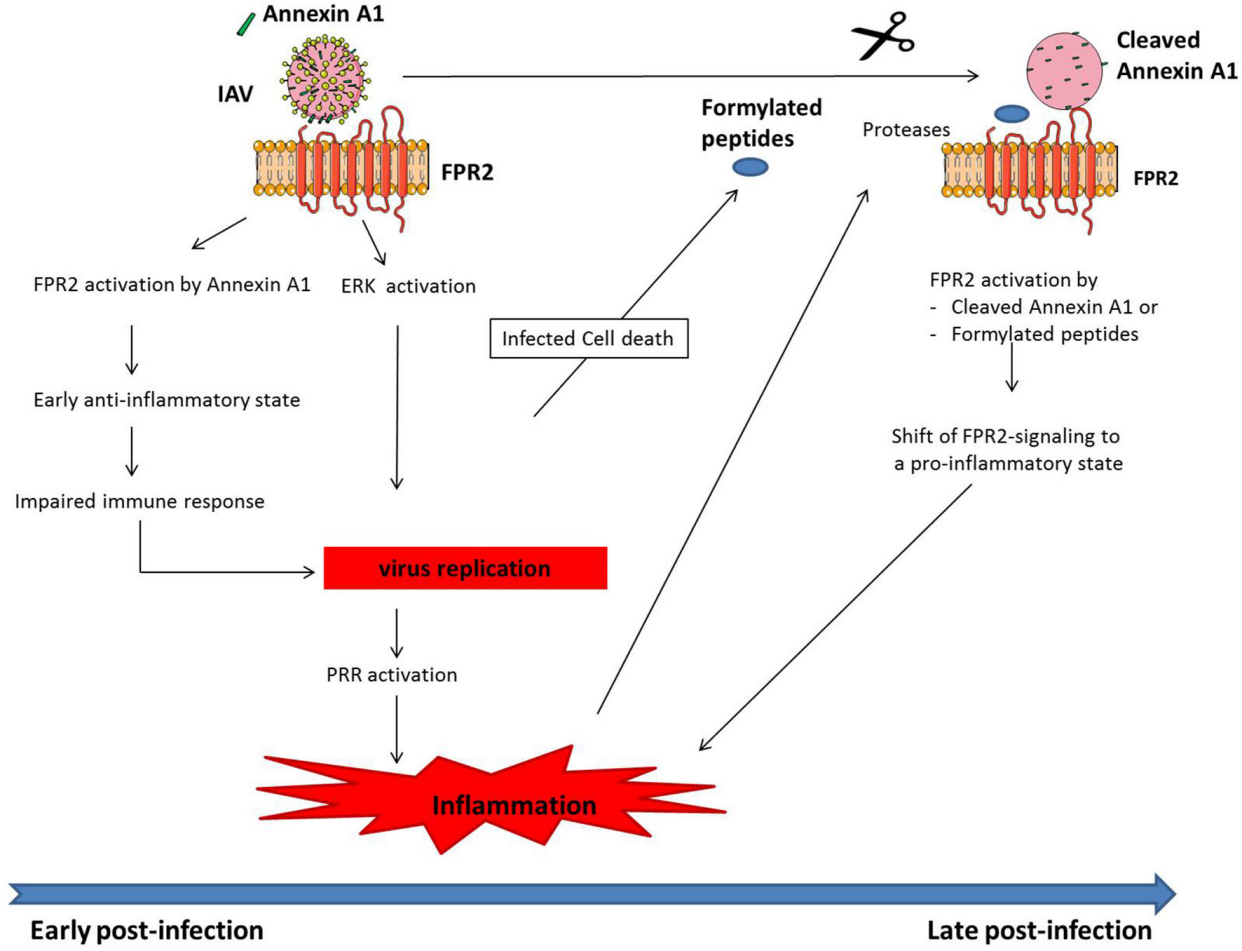
Model of how FPR2 promotes harmful inflammation in a time scale manner. At early stages post-infection, Annexin A1 incorporated into IAV, activates FPR2 leading to (i) an anti-inflammatory state, which impairs host immune response and provides the mean for IAV to replicate. In addition (ii), FPR2-signaling activates the ERK pathway, further increasing IAV replication. Altogether, IAV replication fosters PRR activation, leading to a dysregulated and excessive innate immune response. During the time course of infection, infected cells undergo apoptosis, leading to the release of mitochondrial formylated peptides. In addition, proteases are released in a large amount by leucocytes that are recruited to the site of infection. Those proteases cleave Annexin A1 incorporated into IAV. FPR2, which is then activated by formylated peptides and cleaved Annexin A1 turns pro-inflammatory. This further contributes at later stages post-infection to increased inflammation and IAV pathogenesis.

After IAV infection of a host cell, the virus replicates, and at the end of the viral life cycle, the newly formed virions are released from infected cells. This step occurs through a budding process, in which the virions incorporate cellular plasma membrane proteins, such as AnnexinsA1/A2/A4/A5, glypican 4, CD9 or CD81 or cytoplasmic cellular proteins such as tubulin, enolase 1, actin, tropomyosin 1 and 3, cofilin, cyclofilin or profiling (LeBouder et al., 2008; Shaw et al., 2008; Berri et al., 2014b). Among these proteins, Annexin A1 is of particular interest (Shaw et al., 2008; Tcherniuk et al., 2016). Annexin A1 belongs to the Annexin family of calcium-dependent phospholipid-binding protein and has well known anti-inflammatory functions (Perretti and Dalli, 2009). The upregulation of its expression upon glucocorticoid treatment is one of the mechanisms by which glucocorticoids inhibit inflammation (Perretti and D'Acquisto, 2009). Annexin A1 inhibits leukocyte adhesion to epithelial cells, migration and chemotaxis. As mentioned earlier, the main receptor of Annexin A1 is FPR2. Recently, we have shown that Annexin A1 of IAV binds and activates FPR2 during the adsorption of IAV to a host cell (Tcherniuk et al., 2016). More importantly, upon mouse infection, FPR2 activation is associated with an increase in IAV replication, an exacerbated and harmful pulmonary inflammation and a severe influenza disease (Tcherniuk et al., 2016). Thus, like for major receptors involved in immune signaling pathways, FPR2 is a prototype receptor which is corrupted by IAV for its own benefit by incorporating Annexin A1. In accord with these data, a recent report showed that Annexin A1-deficient mice are protected from IAV replication and virus-induced lethal infections (Arora et al., 2016). Mechanisms through which FPR2 mediates increased viral replication is dependent on the activation of the mitogen-activated protein kinase, ERK (Extracellular signal-regulated kinases), a pathway absolutely required for IAV cell cycle (Pleschka et al., 2001; Droebner et al., 2011; Haasbach et al., 2017). Within the infected cells, FPR2-signaling leads to ERK activation, thus leading to increased virus replication (Tcherniuk et al., 2016). In contrast, the mechanism by which FPR2 promotes severe inflammation during influenza remains to be determined. Indeed, as mentioned earlier, FPR2 is a versatile receptor, acting as pro-inflammatory when activated by formyl peptides but anti-inflammatory when it binds LXA_4_ or Annexin A1. Several hypotheses might explain the failure of FPR2 to restore homeostasis and resolve inflammation to basal levels after acute inflammation (Figure 1). First, since IAV replicates at very high levels when FPR2 is activated, the resulting local necrotic infected cells could generate large amounts of formylated-peptides from mitochondrial proteins. These pro-inflammatory peptides might overcome the anti-inflammatory functions mediated by LXA_4_ / Annexin A1. In this case, FPR2 would turn pro-inflammatory, by binding at later stages of infection to pro-inflammatory ligands. Another possibility is that the initial protective host response to infection required to eliminate the virus is impaired because FPR2 is inadequately activated after infection by IAV-expressing Annexin A1. In consequence, IAV replicates more efficiently, leading to a greater extent activation of PRR and stronger release of pro-inflammatory mediators. In this case, the apparent pro-inflammatory function of FPR2 activation would occur indirectly through increased viral replication. It is also possible that during influenza, Annexin A1 activation of FPR2 leads to pro-inflammatory signaling. Indeed, proteases are present in large amounts at the site of IAV infection and those proteases could cleave Annexin A1, modifying its anti-inflammatory function. Many reports have demonstrated that in contrast to the full length protein, several products of Annexin A1 mediate pro-inflammatory functions, including neutrophil transmigration and leukocytes chemotaxis through FPR2 (Ernst et al., 2004; Williams et al., 2010). In addition, it cannot be excluded that Annexin A1 adopts a specific structural conformation at the surface of influenza virions or heterodimerize in an unusual fashion, thereby promoting FPR2 pro-inflammatory functions. Altogether, although the precise mechanism still remains to be fully determined, FPR2 promotes IAV pathogenesis, through viral replication and dysregulation of the innate immune system. Thus, FPR2 represents an ideal target to treat influenza.

## Antagonists of FPR2 efficacy against influenza *in vitro* and in preclinical studies

To examine the suitability of FPR2 antagonists as a potential novel influenza virus treatment, we have tested several molecules blocking FPR2 function, namely WRW4 (WRWWWW), PBP10 (ten amino acid phosphoinositide-binding peptide, RhoB-QRLFQVKGRR) and BOC-2 (tert-butoxycarbonyle-FLFLF-OH). WRW4 is a six amino acid peptide which specifically impairs FPR2-signaling. It blocks the binding of agonists to FPR2 and thereby its downstream signaling pathway (Bae et al., 2004). PBP10 is a ten amino acid rhodamine-linked peptide which is also highly specific for FPR2. After passing the cell membrane, it binds to phosphatidylinositol 4,5-bisphosphate (PIP2), disturbing actin filaments and blocking FPR2-signaling (Cunningham et al., 2001). In contrast to WRW4 and PBP10, BOC-2 is not a specific antagonist of FPR2. It acts through a competitive inhibition of formyl peptides binding to both FPR1 and FPR2 (Colucci et al., 2011). All three compounds have an antiviral activity in lung epithelial A549cells (Tcherniuk et al., 2016; Courtin et al., 2017). This effect was observed against influenza A subtypes H1N1, H3N2, H6N2 as well as influenza B viruses. Of particular interest, the effect of FPR2 antagonists used in combination with oseltamivir was additive, showing that the combined therapy of FPR2 antagonists with current antiviral drugs is of particular interest. This effect was not surprising given the non-redundant mechanisms of FPR2 molecules (inhibitor of ERK pathway) and oseltamivir (NA inhibitor). *In vivo*, influenza virus-infected mice were protected from lethal infection upon treatment with WRW4 or BOC2. The effect was significant either when the molecules were administered 1 day before infection (prophylaxis), the day of infection or 1/2 days post-infection (treatment). Typically, using a lethal dose of A/PR/8/34 (H1N1) virus infection, 100% of mice reached the experimental endpoints while only 20–40% attained the endpoint after BOC2 or WRW4 treatment (Tcherniuk et al., 2016; Courtin et al., 2017). As expected, this was correlated with a significant inhibition of lung viral titers along with a reduction of the harmful pulmonary inflammation. Interestingly, a previous report showed that cyclosporine A, a specific inhibitor of FPR1 inhibits IAV replication, *in vitro* (Liu X. et al., 2012). Since all FPR have a high degree of sequence homology, these results are consistent with the protective effect of FPR2 antagonists against flu and suggest that other FPR might be involved in IAV pathogenesis. Altogether, these data are a proof of concept that FPR2 antagonists are highly potent novel anti-viral and immunomodulatory agents that could be investigated further to treat influenza virus infections.

## Advantages to treat the flu with FPR2 antagonists with regard to other approaches

Host factors represent useful targets for therapy to overcome the challenge of virus resistance. Some interesting molecules have been identified and this approach appears particularly relevant to treat influenza. The first class of novel promising antivirals are related to their capacity to block cellular functions supporting the virus life cycle. Many targets with antiviral properties were identified, including inhibitors of cytoskeleton, autophagy, proteasome, nuclear export or regulators of transcription (de Chassey et al., 2014). Although these molecules could greatly benefit the development of our arsenal of novel therapeutics, most of them only act on viral replication. Since inflammation is also an important trait of influenza pathogenesis, blocking viral replication would only benefit patients that are treated during the first days of infection.

Another class of molecules aims at the protection of the tissues from damage induced by excessive inflammation. This novel approach concerns mainly all molecules with anti-inflammatory properties. These molecules could benefit patients with severe influenza at later stages post-infection but would not act on viral replication. In this regard, molecules such as statins (Kwong et al., 2009), sphingosine (Teijaro et al., 2011) or anti-platelet drugs (Le et al., 2015) are worth mentioning. These drugs are not expected to be effective when used in prophylaxis or soon after a mild infection.

**Figure 2:**
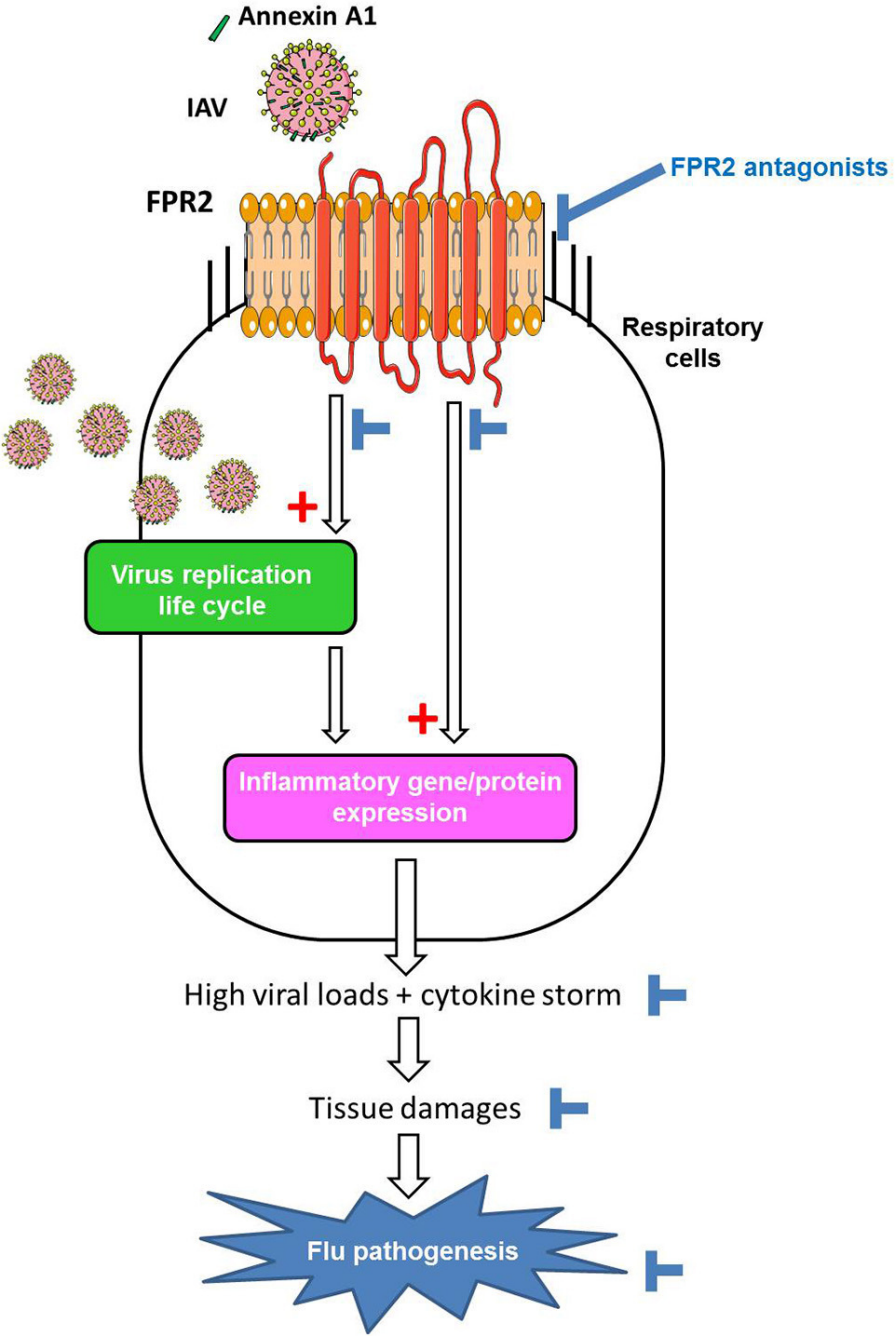
Model of the contribution of FPR2 in influenza virus pathogenesis and effect of FPR2 antagonists. Cellular Annexin A1 incorporated in the envelope of IAV, activates FPR2 during virus absorption to the host cell. FPR2-signaling through the ERK pathways increases infectious virus production (1) contributing to a proinflammatory state via the recognition of viral RNA by PRRs. In addition, FPR2-signaling also directly promotes a pro-inflammatory state (2), by enhancing the release of cytokines/chemokines and impairing the resolution of acute inflammation. Altogether, the excessive recruitment and activation of immune/inflammatory cells contributes to tissue damages and flu pathogenesis. Thereby, by inhibiting virus replication and preventing deleterious inflammation of the lungs, FPR2 antagonists emerge as a novel promising strategy to protect from influenza virus pathogenesis.

In contrast, novel opportunities are currently emerging with the novel class of drugs that both inhibit virus replication and temper inflammation. For example, the antagonists of Protease-activated receptor-1 (Khoufache et al., 2013), calpain proteases (Blanc et al., 2016), NF_*k*_B or ERK (Pinto et al., 2011; Haasbach et al., 2013, 2017), which block viral replication and temper inflammation might be a real opportunity for novel therapeutics against flu. Regarding FPR2, it is also a pivotal receptor involved in IAV replication and harmful inflammation of the lungs during severe influenza (Figure 2). Thereby targeting FPR2 is of particular interest. In addition, although this remains to be investigated, FPR2 is not a critical factor involved in cellular function. Thus, one can expect that FPR2 antagonists will not provide many side effects, in comparison to other targets.

## Conclusion

Preclinical studies have proven that FPR2 antagonists efficiently protect mice against IAV infections, by inhibiting viral replication and deleterious inflammation of the lungs. FPR2 is a host receptor and thus targeting such protein is of particular interest in order to limit the emergence of IAV resistance. In addition, FPR2 antagonists will most likely generate a long lasting protection since it tempers inflammation which is responsible for tissue injury at later stages of the infection. Used together with oseltamivir, FPR2 antagonists might also have a much stronger effect in blocking IAV replication. Altogether by inhibiting viral replication and protecting the lungs from destruction, FPR2 antagonists appear as an appealing strategy to treat or prevent influenza in the future.

## Author contributions

All authors listed, have made substantial, direct and intellectual contribution to the work, and approved it for publication.

### Conflict of interest statement

BR received funds from the patent valorisation SATT-Sud Est organization. The other authors declare that the research was conducted in the absence of any commercial or financial relationships that could be construed as a potential conflict of interest.
